# Stochastic alternative splicing is prevalent in mungbean (*Vigna radiata*)

**DOI:** 10.1111/pbi.12600

**Published:** 2016-08-05

**Authors:** Dani Satyawan, Moon Young Kim, Suk‐Ha Lee

**Affiliations:** ^1^ Department of Plant Science and Research Institute of Agriculture and Life SciencesSeoul National UniversitySeoulKorea; ^2^ Indonesian Center for Agricultural Biotechnology and Genetic Resources Research and DevelopmentBogorIndonesia; ^3^ Plant Genomics and Breeding InstituteSeoul National UniversitySeoulKorea

**Keywords:** alternative splicing, mungbean (*Vigna radiata*), RNA sequencing, stochastic process, evolutionary conservation

## Abstract

Alternative splicing (AS) can produce multiple mature mRNAs from the same primary transcript, thereby generating diverse proteins and phenotypes from the same gene. To assess the prevalence of AS in mungbean (*Vigna radiata)*, we analysed whole‐genome RNA sequencing data from root, leaf, flower and pod tissues and found that at least 37.9% of mungbean genes are subjected to AS. The number of AS transcripts exhibited a strong correlation with exon number and thus resembled a uniform probabilistic event rather than a specific regulatory function. The proportion of frameshift splicing was close to the expected frequency of random splicing. However, alternative donor and acceptor AS events tended to occur at multiples of three nucleotides (i.e. the codon length) from the main splice site. Genes with high exon number and expression level, which should have the most AS if splicing is purely stochastic, exhibited less AS, implying the existence of negative selection against excessive random AS. Functional AS is probably rare: a large proportion of AS isoforms exist at very low copy per cell on average or are expressed at much lower levels than default transcripts. Conserved AS was only detected in 629 genes (2.8% of all genes in the genome) when compared to *Vigna angularis*, and in 16 genes in more distant species like soya bean. These observations highlight the challenges of finding and cataloguing candidates for experimentally proven AS isoforms in a crop genome.

## Introduction

Alternative splicing (AS) is the differential splicing of introns from pre‐mRNA to yield several distinct mature mRNAs (isoforms) from a single gene. In general, four fundamental types of differential splicing can alter the coding region: intron retention, exon skipping, alternative donor and alternative acceptor (Breitbart *et al*., 1987). In intron retention, intron sequences that are normally spliced out are retained in the mature mRNA, producing a longer transcript with extra coding sequences. By contrast, in exon skipping, some exon segments are spliced out from the final transcript to yield shorter mRNA molecules. The location of the splicing reaction can also change at only one of the splice sites; this situation is referred to as ‘alternative donor’ if the change occurs at the 5′ end of the intron and ‘alternative acceptor’ if the change occurs at the 3′ end of the intron.

The mature mRNAs produced by AS can harbour additional bases or lack some exon sequences, resulting in alteration of amino acid composition, physical characteristics or chemical function of the encoded proteins. Thus, AS can increase the number of protein types and phenotypes produced by a small number of genes. Inclusion of additional sequences and mis‐sense splicing can also introduce premature stop codons into the transcripts, making them vulnerable to degradation by the non‐sense‐mediated decay (NMD) pathway (Neu‐Yilik *et al*., 2004) and decreasing the quantity of those particular transcripts in the cell. Several lines of evidence show that cells actually utilize this pathway to modulate and fine‐tune the number of RNA molecules for a particular gene under certain conditions (Filichkin and Mockler, 2012; Kawashima *et al*., 2014).

Consequently, AS could explain the complexity paradox, that is the observation that the genomes of certain complex organisms harbour a smaller number of coding regions than those of some simpler organisms (Graveley, 2001). Several experimentally proven AS isoforms produce multiple proteins with distinct characteristics and function from the same coding region (Inoue *et al*., 1990; Lah *et al*., 2014; Ullrich *et al*., 1995), potentially explaining how a single gene could perform multiple functions in the cell. The advent of next‐generation sequencing (NGS), which can generate large quantities of transcriptome data faster and more cheaply than previous methods, has aided in the identification of AS in many different organisms. Software and script packages, such as ASTALAVISTA (Foissac and Sammeth, 2007) and ASprofile (Florea *et al*., 2013), have been developed to rapidly identify splicing variants by examining variations in exon–intron boundaries in genomewide alignment data generated using NGS. The results are quite surprising: in some cases, AS occurred in more than half of the annotated genes (Marquez *et al*., 2012; Pan *et al*., 2008; Shen *et al*., 2014). If all AS produces functionally divergent proteins, then this process regulates the bulk of transcript generation and protein synthesis in the cell. Hence, proper annotation of the occurrence of AS in the genome is very important as a reference for functional genomic studies.

Several studies have attempted to catalogue the global occurrence of AS in the genomes of several plants, including soya bean (Shen *et al*., 2014), *Arabidopsis* (Filichkin *et al*., 2010; Marquez *et al*., 2012) and maize (Thatcher *et al*., 2014), by utilizing mRNA sequences obtained from different tissue types under diverse environmental conditions to capture as many transcript types as possible. Nevertheless, although those studies revealed that a large number of plant genes undergo AS, very little experimental evidence of functional AS proteins is available (Severing *et al*., 2009). Several groups have suggested that the scarcity of demonstrably functional AS isoforms could be due to the random nature of AS itself (Hon *et al*., 2013; Melamud and Moult, 2009a; Zhang *et al*., 2009), implying that most AS isoforms have no function because they are merely the by‐products of erroneous splicing. Consequently, it is unlikely that all AS events are part of a distinct layer of gene regulation. That said, because AS isoforms that confer selective advantage could be retained by progeny with stronger AS signals for those isoforms, functional AS could still evolve and be retained by natural selection.

Because advantageous AS isoforms have a higher probability of being retained over the course of evolution, it should be possible to identify them in comparative studies of related species. Mungbean (*Vigna radiata*) and its close relatives in the *Vigna* genus, like adzuki bean (*Vigna angularis*), are good candidates for such studies. Their genome sequences have recently been published (Kang *et al*., 2014, 2015), enabling transcript alignment and facilitating identification of AS isoforms. They are also related to soya bean, whose genome is already well characterized, and for which comprehensive data regarding AS are available. Because mungbean and adzuki bean are widely planted for food consumption (annual plantation area of 6 million and 840 000 hectares, respectively), any practical applications that could be derived from genomic studies in these plants will have considerable economic impact (Nair *et al*., 2012; Rubatzky and Yamaguchi, 1997).

We performed global transcriptome analysis to identify and catalogue AS events that occur in mungbean. To infer the characteristics of AS regulation in this species, we tested for stochastic AS in the RNA population. To identify AS events with the strongest likelihood of being functional, for the purpose of subsequent in‐depth studies, we investigated AS conservation in adzuki bean and soya bean. The resultant whole‐genome annotation of AS isoforms represents a valuable contribution to the mungbean genome sequencing project.

## Results

### Characteristics of AS types in mungbean

The number of AS events (hereafter, AS number) of each type were detected *in silico* based on alignment of RNAseq data to the mungbean reference genome. Shotgun sequencing generated, on average, 38.6 million 100 bp reads per sample (Table S1), close to 10 times the size of the mungbean genome. The total length of annotated transcribed regions is 104 million bases; therefore, the sequence alignment produced roughly 37× sequencing coverage for all open reading frames. However, because most of the sequenced RNAs are derived from mature RNAs whose introns have been spliced out, the sequencing depth in exonic regions was 101× on average.

The number of AS events detected varied with the software pipeline: ASprofile annotated more AS events than ASTALAVISTA (Table 1). A closer inspection of the binary alignment (BAM) files using a genome browser revealed that the higher number of AS events detected by ASprofile was due to reporting of new exons not found in the mungbean genome annotation, as well as increased sensitivity in detecting rare splicing junctions. Depending on the AS types, ASTALAVISTA did not detect 85.3–90.1 per cent of AS events detected by ASprofile. However, ASprofile also failed to detect 56.5–85 per cent of AS events detected by ASTALAVISTA. Neither pipeline is clearly superior to the other as they both missed AS events detected by the other pipeline, but ASprofile output was used for further analysis due to its increased sensitivity and better annotation system.

**Table 1:** Number of AS isoform types in each tissue, based on isoform detection with ASTALAVISTA and ASprofile

Detection method	Tissue	Intron retention	Exon skipping	Alternative donor	Alternative acceptor	Affected genes
ASTALAVISTA	Flower	3620	659	1141	2101	4414
	Leaf	3512	647	1107	2060	4256
	Pod	4076	589	1093	2132	4541
	Root	5419	772	1320	2401	5429
ASprofile	Flower	4256	6494	3373	3059	8051
	Leaf	4173	6185	3278	2897	8152
	Pod	4582	6394	3455	3103	7931
	Root	5874	6724	3885	3336	7549

ASprofile estimated that 44.6% of mungbean genes are subjected to AS, whereas ASTALAVISTA estimated this proportion as 37.9%. Both figures are lower than the proportions reported for *Arabidopsis* (Marquez *et al*., 2012), maize (Thatcher *et al*., 2014), rice (Lu *et al*., 2010) and soya bean (Shen *et al*., 2014), but fairly similar to those of closely related legumes like *Medicago* and *Phaseolus* (Chamala *et al*., 2015).

**Figure 1:**
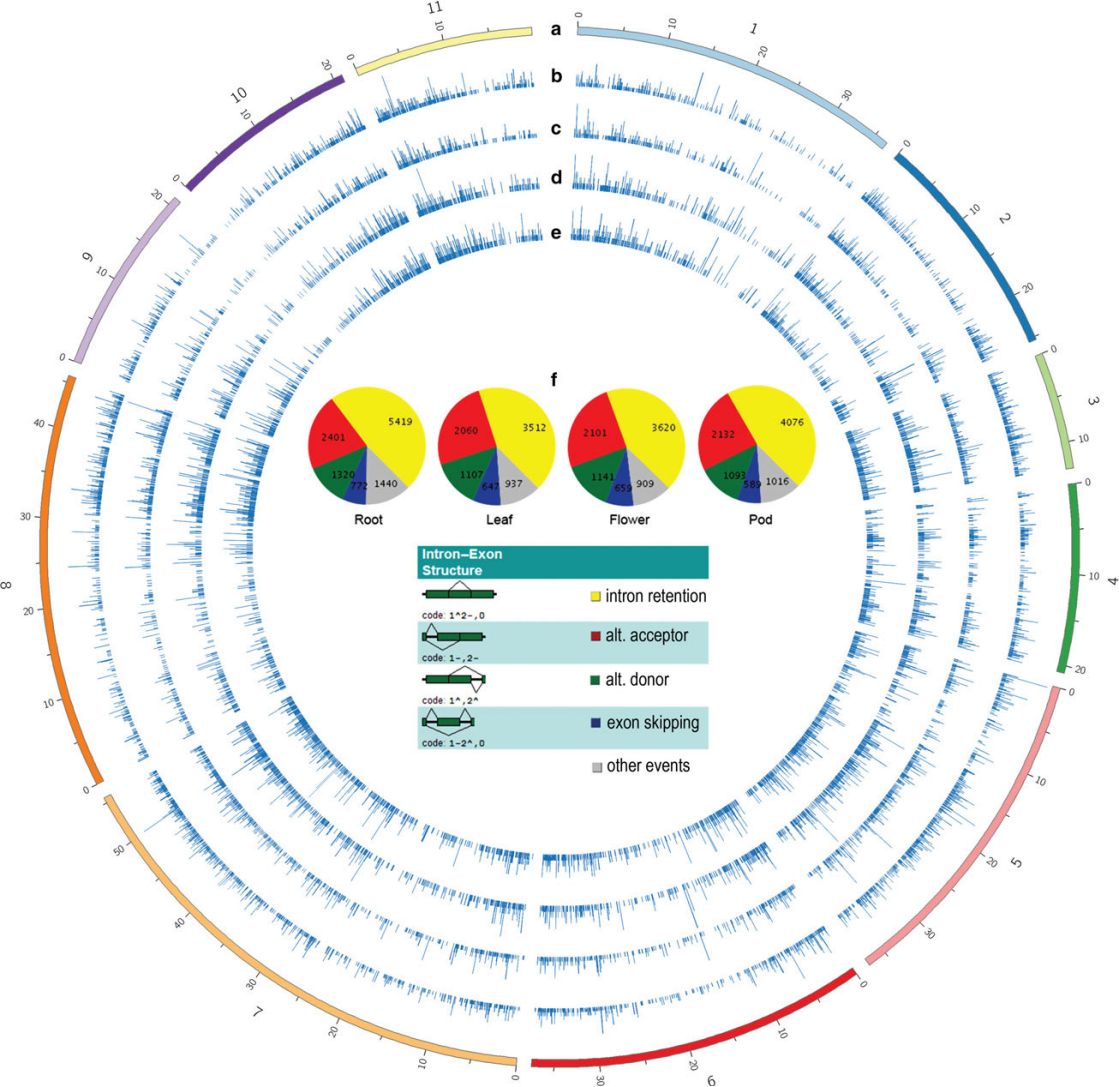
Types and chromosomal distribution of AS in four mungbean tissues. From outer to inner rings: (a) size of chromosome (in megabases); (b) histogram of AS number across chromosomes in root, (c) leaf, (d) flower and (e) pod tissues. (f) Proportions of each type of AS across the four tissues, as classified by ASTALAVISTA.

The distribution of AS was generally similar across tissues (Figure 1), although tissue‐specific AS isoforms were detected, and various tissues yielded different numbers of AS isoforms. Among all tissues, roots had the highest number of AS events, as well as the highest AS number per gene (Table 1). One potential reason for this is that roots express the largest number of tissue‐specific genes, and these genes tend to be highly expressed (Table S2), potentially aiding in detection of AS isoforms in RNA from roots. About 2.3% of tissue‐specific AS events were the consequence of tissue‐specific gene expression, and their absence in other tissues is caused by the lack of expression of those genes; however, the remaining AS isoforms are tissue‐specific even though the originating transcripts are expressed at significant levels in more than one tissue type. In many cases, we found that the absence of AS isoforms in other tissues was not merely caused by low expression levels and under‐representation in RNA samples from these tissues.

The number of transcripts representing a particular isoform is difficult to quantify accurately without long‐read sequence data, because some genes have multiple AS events and some isoforms may combine with others from the same gene to generate a distinct transcript structure. Because long‐read RNAseq data were not available for this study, we simply assumed that such combinations were nonexistent and then estimated the quantity of each AS isoform based on the number of sequence fragments that aligned to the splice junction that underwent AS. Based on this assumption, a significant portion of detected AS isoforms (20.54%) had FPKM values lower than 1, that is their concentration is very low in an average cell. Moreover, a considerable proportion of AS isoforms (24.4%) were expressed at levels <10% of those of the more abundant constitutive splice forms.

### Mungbean AS exhibits signs of stochastic splicing

**Figure 2:**
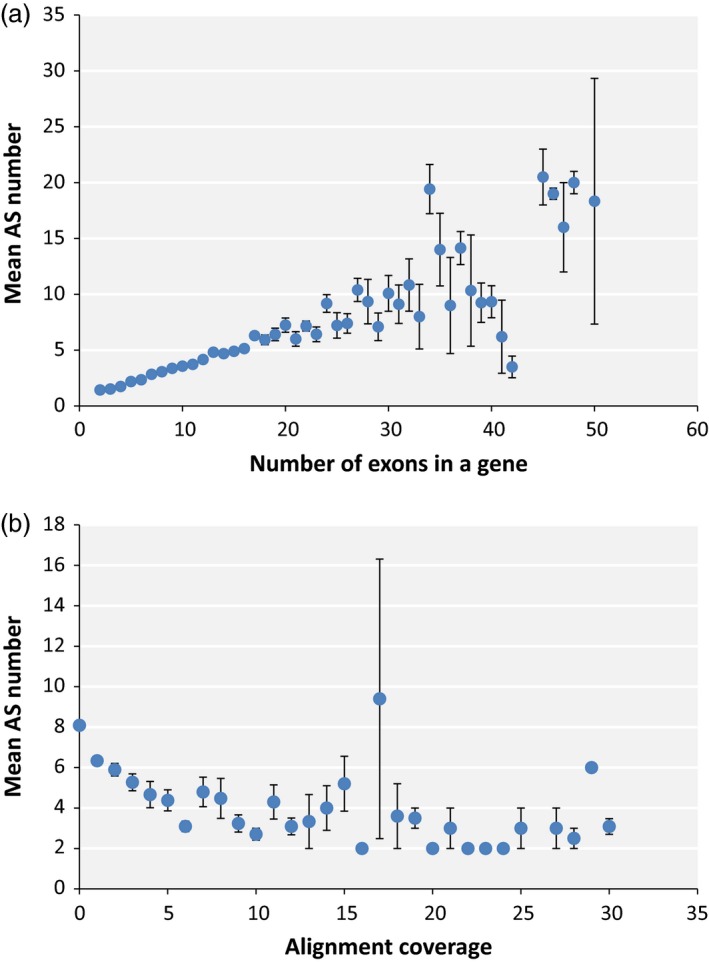
Correlation of mean AS number with number of exons in a gene (a) and gene expression level estimated from alignment coverage (b).

**Figure 3:**
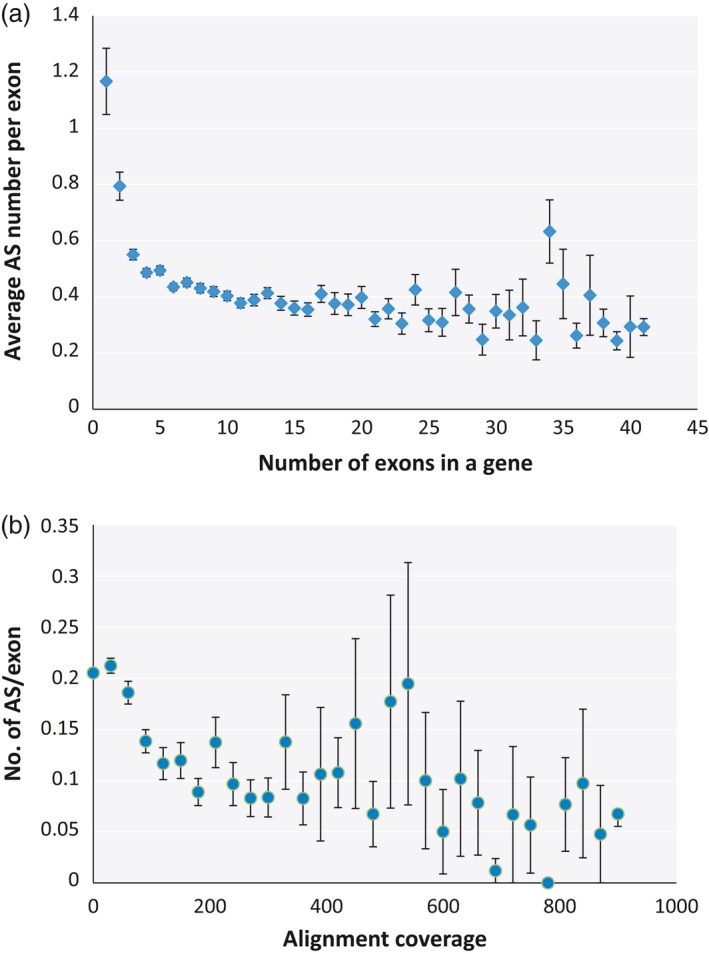
Comparison of the average number of AS events per exon, calculated by dividing the number of AS events in a gene with the number of exons in that gene, for genes containing different numbers of exons (a) and genes expressed at different levels (b), as determined by alignment coverage per base.

The prevalence of AS isoforms with low concentration in our mungbean AS data raises the possibility that a significant number of mungbean AS could be the result of random errors with little effect on the protein composition of the cell. To determine whether stochastic splicing is prevalent in mungbean, we investigated the correlation between the presence of AS and several aspects of the plant's genomic features that may increase the probability of random splicing errors. We found that mean AS number was strongly correlated with the number of exons in a gene with a Pearson *r*‐value of 0.879 and *P*‐value of 4.09e‐14 (Figure 2a). This is consistent with the random splicing error model: the higher the exon number, the larger the number of splicing junctions and the greater the chance of error associated with splicing of those junctions. However, an obvious consequence of probabilistic splicing error is that genes with a large number of introns will have a higher probability of accumulating useless splicing errors, which could be dangerous to the cell. To determine whether mungbean has evolved a mechanism to reduce the likelihood of such errors, we plotted the average number of AS per exon for genes with different exon numbers. The plot reveals a clear trend towards fewer AS events per exon as the number of exons increases (*r* = −0.649, *P* = 7.755e‐05), although the pattern is less clear for genes containing more than 25 exons (Figure 3a).

Erroneous splicing is also more disadvantageous for highly expressed genes, because in such cases, it would create a large amount of mis‐spliced mRNA, which in turn is more likely to be translated into a large quantity of nonfunctional protein. Consistent with this, we observed a trend towards fewer AS events per exon in highly expressed genes (Figure 3B), although the correlation was weak (*r* = −0.057 and *P*‐value = 2.2e‐16). A correlation plot of AS number vs expression level revealed a negative correlation (*r* = −0.463 and *P*‐value = 0.001) between the two variables (Figure 2b). This observation contrasts with findings in other plants such as soya bean, in which highly expressed genes also usually have higher numbers of AS events (Shen *et al*., 2014). One reason for this could be that, in mungbean, the average number of exons is lower among highly expressed genes (Figure S1), and exon number correlates more strongly to AS number than expression level. Moreover, the average number of AS was not higher in highly expressed genes than in genes carrying the same exon numbers expressed at lower levels (Figure S2).

**Figure 4:**
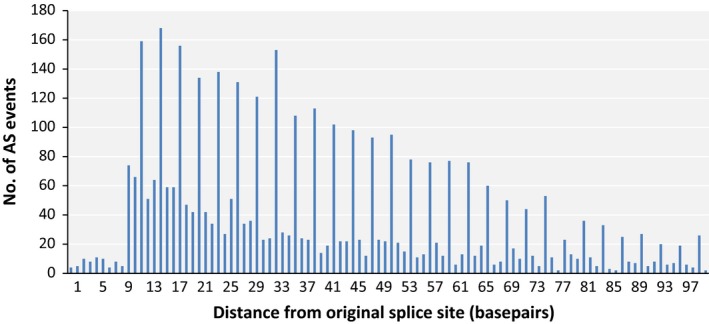
Number of AS isoforms, according to distance from the regular splice site, for alternative donor and alternative acceptor isoform types.

Another important effect of AS on the final transcript sequence is the creation of frameshift mutations, which could significantly alter the amino acid composition downstream of the splice site. Splicing error can introduce or eliminate *n* + 1, *n* + 2 and *n* + 3 nucleotides to the mature mRNA, where *n* is a multiple of three nucleotides and only the *n* + 3 variant will preserve the downstream codons. Assuming that all three variants are equally likely to occur, random splicing error should introduce frameshift 67% of the time. Frameshift mutations have the highest probability of rendering the resulting protein nonfunctional; therefore, we were curious to see whether this phenomenon would be repressed in mungbean AS. Frameshift formation is close to 67% for AS events of the exon skipping and intron retention types (Table 2). However, the creation of frameshift was lower than expected in the alternative donor and alternative acceptor types of AS: plotting the number of AS events that occurred several bases from regular splicing sites revealed a preference for multiples of 3 (i.e. the length of a codon) in these types of AS (Figure 4). This could be partially explained by the common occurrence of NAGNAG motifs near the 3′ end of introns (Shi *et al*., 2014). Because plant splice sites are normally located at AG bases at the 3′ ends of introns, such motifs would direct spliceosomes that miss their target to alternative targets located at distances that are integral multiples of codon lengths, thereby preventing the formation of frameshifted mRNA. Curiously, this effect was still visible at positions as far as 90 bases from the regular splice sites, distances at which NAGNAG motifs are unlikely to persist. Because frameshift mutation is very common in the more abundant intron retention and exon skipping types, it is unlikely that this is mostly caused by natural selection against frameshift splicing; therefore, other mechanisms could be responsible for these effects.

**Table 2:** Proportion of AS isoforms carrying frameshift codons in the mature mRNA, classified according to AS types

Type of AS	Root (%)	Leaf (%)	Pod (%)	Flower (%)
Alternative donor	34.3	33.9	32.6	29.9
Alternative acceptor	46.3	42.1	42.3	41.7
Exon skipping	72.0	72.5	71.2	72.2
Intron retention	61.6	63.2	62.9	62.6

### The role of sequence variation and the extent of AS conservation

**Figure 5:**
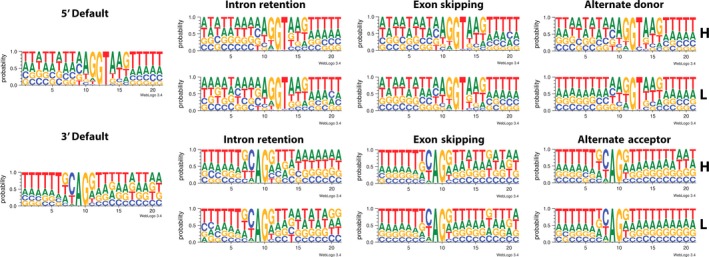
Proportion of DNA bases surrounding alternative splice types compared to the regular splice sites. AS isoforms with FPKM >10 were categorized as having high concentration (**H**), while those with FPKM <10 were grouped into the low concentration group (**L**).

The presence of motifs like NAGNAG at the 3′ ends of introns raises the question of whether AS sites occur only at canonical splice sites or utilize other bases as well. We surveyed all splice junctions of the default and AS isoforms and categorized each AS site as high or low, using FPKM value of 10 as a threshold. At the 3′ ends of introns, all isoforms utilize the AG splice site; by contrast, at the 5′ end, a majority of splice sites occur at GU bases but a small fraction also occur at GC bases (Figure 5). There were no obvious sequence patterns around the two bases that could explain why some AS sites are more frequently spliced than others. Hence, although the pattern of AS appears random, it is still constrained by the availability of bases that can be used as splice sites. However, because the required motifs at each end are only two bases long, and the abundance of these dinucleotides in the genome is relatively high, it is not surprising that AS is so prevalent in many organisms.

Nevertheless, functional AS isoforms have been detected in the past (Inoue *et al*., 1990; Ullrich *et al*., 1995); hence, it is not unlikely that mungbean also harbours some functional isoforms in its transcriptome. We tried to identify candidate AS isoforms for more in‐depth study of their function. However, given that a significant portion of AS in mungbean may not have any function at all, we paid extra attention to AS sites that are conserved in other species. Conservation among species does not necessarily imply function, but it at least indicates that the isoforms in question do not impose negative selection pressure on the plant over evolutionary timescales. To this end, we compared transcript sequences from mungbean to those from adzuki bean (*Vigna angularis*), a closely related species in the *Vigna* genus. BLAST analysis of exon sequences surrounding AS junctions identified 3600 AS sites with high sequence similarities in both species (Table S3), which is comparable to the results obtained by Chamala *et al*. (2015), who identified more than 5000 conserved AS between common bean and soya bean using a similar method. However, a closer examination also revealed that the exact splice sites are rarely conserved in both species. By applying the strict criteria that both splice sites must be located at the exact same position and the differences in nucleotide length between the two species must not introduce frameshift, we reduced the set of candidates to 629 genes, comprising 859 conserved AS events (Table S4).

**Figure 6:**
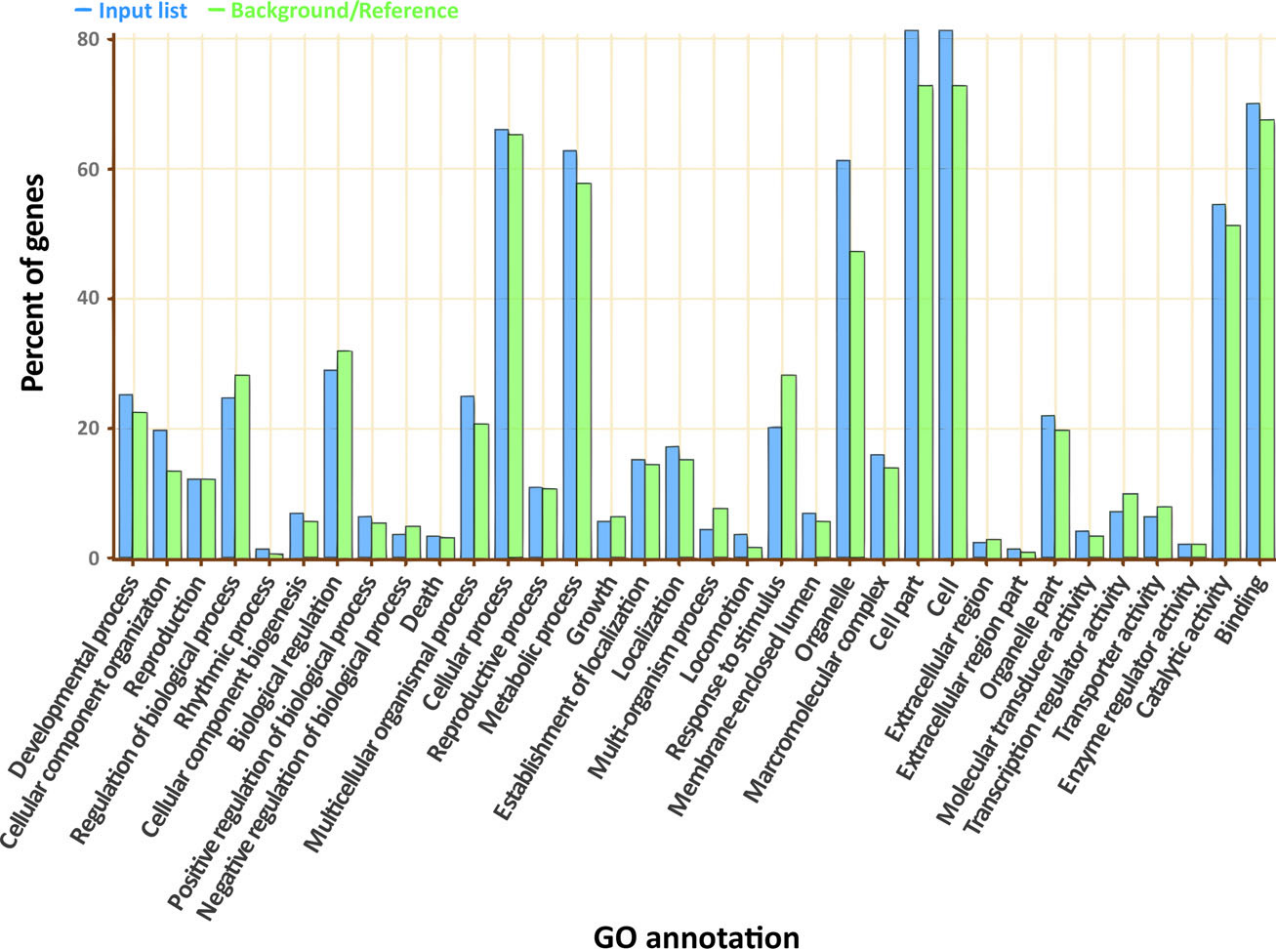
Gene ontology enrichment of genes with AS isoforms that are conserved in mungbean and adzuki bean.

Gene Ontology (GO) analysis of the genes carrying conserved AS identified 488 GO groups (Table S5) with significant enrichment for cellular components only (Figure S3), while other GO groups are not significantly different from background level (Figure 6). However, the number of conserved AS isoforms dwindles even further when the comparison is made between more distantly related species. A comparison of AS events between mungbean and soya bean (Glycine max) yielded only 16 conserved AS isoforms retained at the exact same base position in both species (Table S6). All but one AS junction was also conserved in adzuki bean, although two of them will create frameshift mutations in adzuki bean. Based on this observation, we conclude that AS events that confer selective advantages, and are thus retained over evolutionary timescales, are very rare. However, other groups have observed that when the conservation criteria are relaxed to ignore the exact splicing position and focus on exon sequence conservation among the same AS types, the number of conserved AS events increases considerably, and such events can be identified even in species outside the angiosperms (Chamala *et al*., 2015). While it is possible that such approach could identify conserved AS with similar function, it would be inadequate to identify possible inclusion of frameshift caused by differences in nucleotide length among species, which can create a very different protein if the isoforms are translated.

## Discussion

Based on the findings in this study, we conclude that the noise hypothesis fits the pattern of AS events in mungbean; consequently, a large proportion of AS isoforms in mungbean probably have no function. However, the noisy splicing model does not exclude the possibility that useful and functional AS isoforms could emerge among the resultant nonfunctional isoforms. As suggested by our observations regarding genes with high exon numbers or expression levels, natural selection will act on genes that produce AS isoforms at concentrations that could be dangerous to the cell, possibly by favouring the propagation of individuals with stronger splicing signals at the correct bases, or those that lack the bases that are used as AS sites. Similarly, AS isoforms that confer selective advantage will be retained or even strengthened, so that the spliceosome will regularly cut at the alternative site as well as the regular site. The presence of numerous low‐abundance isoforms is probably not significantly harmful to mungbean cells, but it could be useful as a source of variants upon which natural selection can act.

The arguments in support of noisy splicing have been outlined by several groups (Hon *et al*., 2013; Pickrell *et al*., 2010; Zhang *et al*., 2009). Melamud and Moult (2009b) noted that while some tissue‐specific AS isoforms are conserved across species, these represent a relatively small fraction of AS events. A large proportion of AS isoforms also carry premature stop codons, which make them vulnerable to NMD. Even if these isoforms are translated, most of the alternative protein structures are predicted to be nonviable. The number of detected AS isoforms also tends to increase in genes with more introns or genes expressed at higher levels, in line with the view of AS as a probabilistic event. The more the introns to be processed, the greater the probability of noisy splicing. Similarly, higher levels of gene expression increase the likelihood of a splicing error among the pool of processed transcripts. This could explain why RNAseq data typically allow the detection of more AS isoforms, because the sequenced libraries usually have a very high sequencing depth. Thus, splicing errors that are not normally found in average cells become visible; this is compounded by the use of PCR during library preparation, which could amplify uncommon transcripts to a more easily detectable level.

Protein studies provide another line of evidence supporting noisy splicing. In human cells, the observed protein diversity revealed by high‐throughput mass spectrophotometry is much lower than that predicted from AS studies of transcriptome data. Abascal *et al*. (2015) found that most human proteins exist as single dominant isoform and detected only 282 AS isoforms among 12 716 genes at the protein level; this is nowhere near the prediction of 95% based on transcriptome data (Pan *et al*., 2008). However, the absence of protein products of a given AS isoform may not necessarily mean that the isoform serves no function; in some cases, degradation of AS isoforms through the NMD pathway serves to regulate the concentration of transcripts in the cell (Drechsel *et al*., 2013; Fu *et al*., 2009). Nevertheless, this lack of representation at the protein level undermines the idea that AS increases the protein diversity generated by a given number of genes.

On the other hand, several compelling arguments support the notion that AS plays an important regulatory role in the cell. Barbazuk *et al*. (2008) presented several lines of evidence for the functional importance of AS: the predominance of AS in some gene families versus its absence in others; the existence of AS events that correlate with specific tissue and developmental cues; incorporation of AS products into ribosomes; and conservation of some AS events among distantly related species. Because noisy splicing has probably existed since the emergence of introns in eukaryotes, it is likely that a large number of useful isoforms have evolved from it, resulting in the phenomena detailed above. However, based on the observed low level of conservation among species, the contribution of AS to protein variation and regulation of gene expression does not appear to be significant.

The actual proportion of functional AS isoforms in mungbean obviously cannot be precisely determined without experimental tests. However, in general the number of AS isoforms is lower in plants than in animals (Kim *et al*., 2008). It will be interesting to speculate whether this is due to the different research focus in plants and animals, or instead to intrinsic differences in the splicing mechanisms in the two kingdoms. One issue that should be investigated is the effect of genome expansion (e.g. polyploidization) on AS. An organism whose genome cannot tolerate significant expansion will benefit greatly by the ability to increase the functionality of its existing genome via AS. Hence, to generate additional transcript diversity, it may be advantageous to maintain a less‐specific splicing machinery. However, polyploid plants can easily obtain new gene variants by allowing duplicated genes to evolve independently; this strategy is potentially safer because it does not disrupt the function of the original gene. In allotetraploid soya bean, duplicated genes undergo less AS (Shen *et al*., 2014); this is curious because in such cases, the penalty for incorrect splicing would be less severe because a backup copy is present elsewhere in the genome.

Because examples of functional AS in plants are rare, it would be prudent to assume that a significant portion of AS in plants has no distinct function; thus, more evidence is required to support the claim that there is extensive functional diversity generated by AS in plants. Thus, detected AS events should be treated like genetic marker data, which are useful to identify and catalogue, but strong experimental evidence such as QTL mapping and transgene expression are necessary to assert that a given sequence variation causes a particular phenotype. Additionally, approaches like comparative genomics, which are used to select candidate markers that are likeliest to alter a phenotype, could also be applied to finding AS isoforms that encode a novel function. We believe that our comparative AS detection data could be used as a starting point to perform more in‐depth studies of the phenotypic diversity generated by AS.

## Experimental procedures

### Plant materials and RNA sequencing

RNA sequence data were obtained from Kang *et al*. (2014); in that study, a pure line mungbean plant from cultivar VC1973A (developed by AVRDC) was used as the source material for RNA extraction. The plants were planted in a greenhouse; following sowing, tissues were harvested from root after 2 weeks, leaf after 1 month, flower after 2 months and whole pods after 2.5 months.

### Sequence alignment, transcript assembly and AS identification

The cleaned sequence data were aligned to the mungbean reference genome (Kang *et al*., 2014) using TopHat (Trapnell *et al*., 2009) with default settings. The resultant gapped alignment data in binary alignment format were then used as input for Cufflinks (Trapnell *et al*., 2012) under default settings to assemble the transcripts and identify splicing junctions from the alignment data. For AS detection and annotation, the assembled transcriptome files (in.gtf format) were submitted to ASTALAVISTA (Foissac and Sammeth, 2007) web interface (http://genome.crg.es/astalavista/). AS events were also annotated with ASprofile (Florea *et al*., 2013), which also uses Cufflinks output as input data. The resulting AS annotations were checked at random by visual examination of the AS genome coordinates in the original binary alignment (.bam) files using Integrated Genome Viewer (Robinson *et al*., 2011).

### Isoform quantitation

The FPKM (fragments per kilobase of transcript per million mapped reads) value for each AS isoform was provided by ASprofile, based on Cufflinks estimation, after assembly of each transcript. When the FPKM value was not available for a chromosomal segment of interest, transcript quantity was estimated based on alignment coverage on that segment, calculated using the coverageBed command in Bedtools (Quinlan and Hall, 2010). The number of aligned fragments was then multiplied by fragment length and divided by the number of bases in the segment of interest, to yield the average coverage per base value. Because each tissue had different sequence coverage, coverage per base was only compared within a tissue.

### Statistical analysis

Basic arithmetical analysis, such as calculations of sums and means, was performed in Microsoft Excel. Calculation of descriptive statistics was performed in the R statistical package, using the ‘describeBy’ command in the psych library. Calculations of Pearson's correlations and the corresponding significance values were also carried out in R using the ‘cor.test’ command.

### Sequence junction analysis

To visualize the presence of sequence conservation near splice sites, coordinates of splice sites along with 10 bases upstream and 10 bases downstream from those sites were input into Bedtools using the fastaFromBed command to obtain the DNA sequences between those coordinates. Sequences with FPKM value >10 were put in the high group, while the rest were put in the low group. The sequences were then used as input for weblogo (http://weblogo.threeplusone.com/) to visualize the proportion of bases commonly found surrounding the splice sites.

### Comparative analysis

The sequence of 50 bp of exonic region surrounding AS sites and intron sequences from intron retention AS events were obtained using Bedtools from mungbean, adzuki bean (*Vigna angularis*) and soya bean (*Glycine max*). Splice sites were detected in adzuki bean and soya bean using ASprofile with the same settings as used for AS detection in mungbean. The RNA sequences used for AS detection were obtained from sequences provided by Kang *et al*. (2015) for adzuki bean and Shen *et al*. (2014) for soya bean. Sequences that share similarities were detected using local BLAST+ search (Camacho *et al*., 2009), with mungbean sequences used as the local sequence database. The resulting matches were filtered using the following criteria: sequences at the splicing junctions must have exact match while sequences further away are allowed to have gaps and mismatches, intron sequences in intron retention type have at least 80% similarities, and the length difference of retained introns and skipped exons between two species must not exceed 30 nucleotides or introduce frameshift. Full sequences of proteins containing conserved AS were then identified, and their homologs in soya bean were identified using blastp in the BLAST+ package. Matching soya bean gene ID with the highest e‐values were then submitted to agriGO (http://bioinfo.cau.edu.cn/agriGO/analysis.php) to obtain the gene ontology classification of those genes.

## Supporting information


**Figure S1.** Average number of exons in a gene, with genes grouped by expression level.
**Figure S2.** Average number of AS events (*y* axis) in genes grouped by expression levels (*x* axis), depending on the number of exons found within the genes.
**Figure S3.** AgriGO annotation of the relationships and significance level of enriched GO groups of the genes with conserved AS in mungbean and adzuki bean. Inside the boxes: numbers inside the brackets are the *P*‐value, while the numbers on lower left sides are annotated/total number in query and on the lower right are annotated/total number in background/reference.

**Table S1.** Sequencing data and mapping rates in each tissue, as summarized by samtools flagstat.
**Table S2.** AS and expression levels of genes expressed in only one tissue type. Expression level is calculated using transcript per million (TPM) method.
**Table S3.** AS types with conserved exon sequences around the splicing junction in both mungbean and adzuki bean.
**Table S4.** Genes carrying AS conserved in mungbean and adzuki bean, along with their encoded protein as listed in mungbean genome annotation.
**Table S5.** Gene ontology terms of the soybean homologs of genes with conserved AS in mungbean and adzuki bean.
**Table S6.** Types and locations of conserved AS in mungbean and soybean, identified using blast alignments of exons and introns surrounding AS sites.
