# Supplementary material for: Stochastic alternative splicing is prevalent in mungbean (Vigna radiata)
Source: Plant Biotechnol J. 2016 Aug 5;15(2):174–82. doi: 10.1111/pbi.12600 (PMC5258860; doi:10.1111/pbi.12600)
Supplement: Supplementary file 1 — Figure S1. Average number of exons in a gene, with genes grouped by expression level. Figure S2. Average number of AS events (y axis) in genes grouped by expression levels (x axis), depending on the number of exons found within the genes. Figure S3. AgriGO annotation of the relationships and significance level of enriched GO groups of the genes with conserved AS in mungbean and adzuki bean. Inside the boxes: numbers inside the brackets are the P‐value, while the numbers on lower left sides are annotated/total number in query and on the lower right are annotated/total number in background/reference. [file PBI-15-174-s001.docx]

**
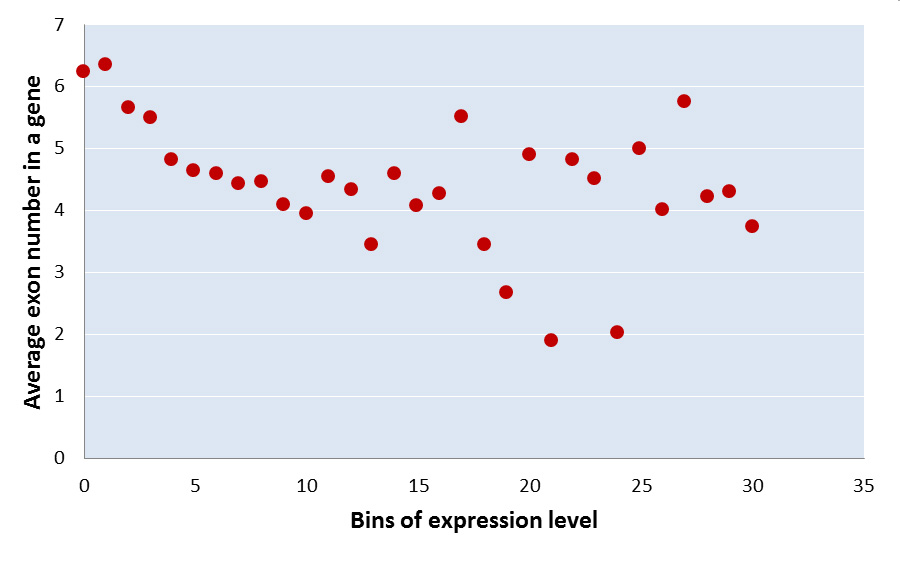
Figure S1.** Average number of exons in a gene, with genes grouped by expression level.

**
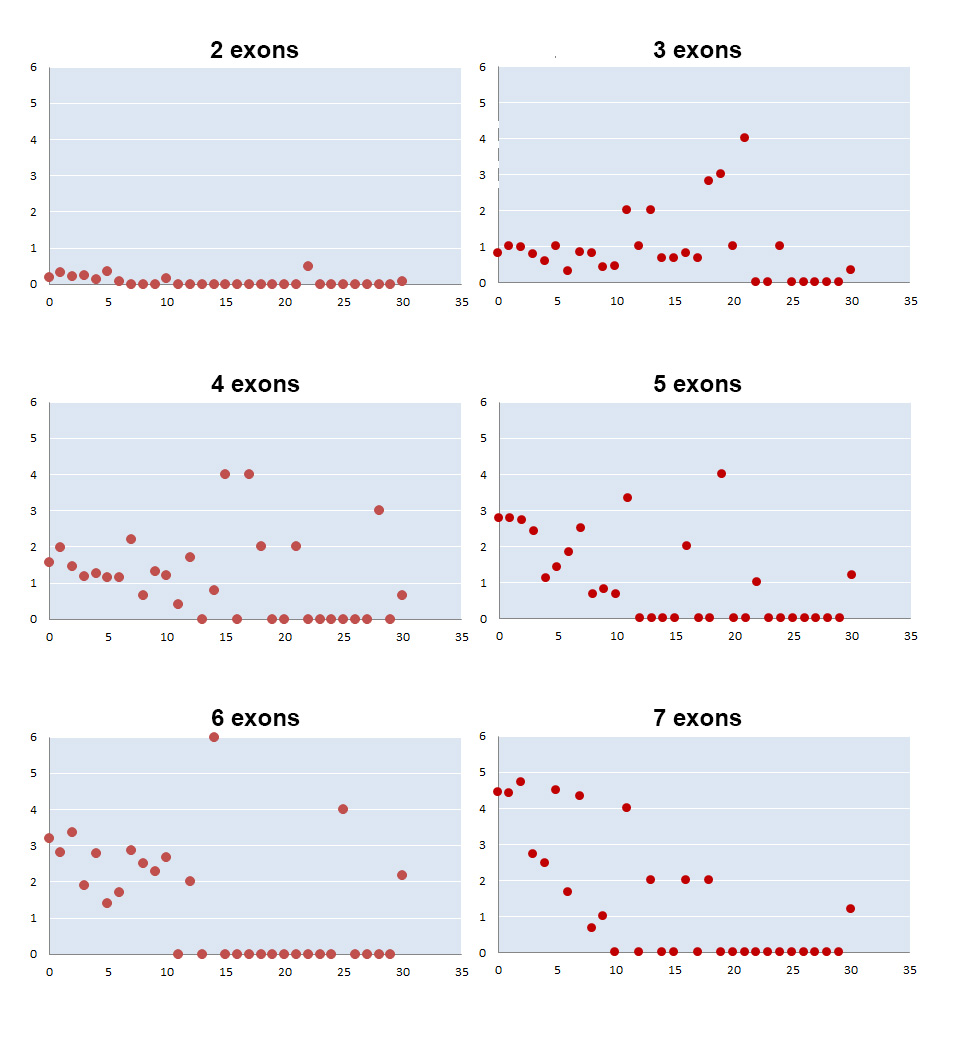
**

**Figure S2**. Average number of AS events (y axis) in genes grouped by expression levels (x axis), depending on the number of exons found within the genes.


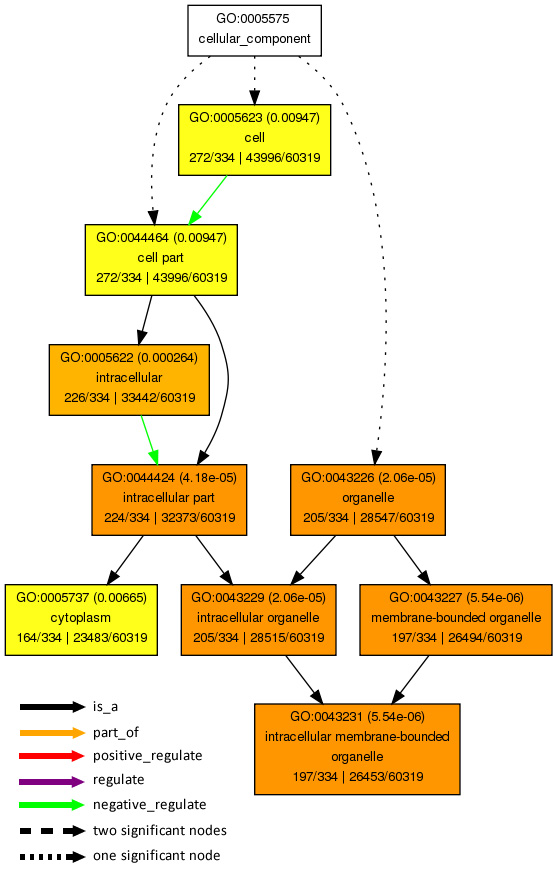


Figure S3. AgriGO annotation of the relationships and significance level of enriched GO groups of the genes with conserved AS in mungbean and adzuki bean. Inside the boxes: numbers inside the brackets are the p-value, while the numbers on lower left sides are annotated/total number in query and on the lower right are annotated/total number in background/reference.
